# Whole genome sequencing data of *Vigna nakashimae* var. Ukushima and G418

**DOI:** 10.1016/j.dib.2020.105131

**Published:** 2020-01-14

**Authors:** Eri Ogiso-Tanaka, Sompong Chankaew, Takehisa Isemura, Alisa Kongjaimun, Akiko Baba, Ken Naito, Akito Kaga, Norihiko Tomooka

**Affiliations:** a Genetic Resources Center, National Institute of Agrobiological Sciences (NIAS), 2-1-2 Kannondai, Tsukuba, Ibaraki 305-8602, Japan; b Program in Plant Breeding, Faculty of Agriculture at Kamphaeng Saen, Kasetsart University, Kamphaeng Saen, Nakhon Pathom 73140, Thailand; c Research Fellow of the Japan Society for the Promotion of Science, Japan

**Keywords:** *Vigna nakashimae*, Closely wild relatives of Adzuki bean, Next-generation sequencing, HiSeq, Whole genome sequencing

## Abstract

*Vigna nakashimae* is one of the closely related species of *Vigna angularis* (Adzuki bean). Two strain of ‘Ukushima’ and ‘G418’ were identified as salt tolerance strains in *Vigna nakashimae* from gene bank collection. F_2_ populations from an inter- or intra-specific cross between the sensitive and tolerant strains are useful for the detection of salt tolerance QTL in *Vigna nakashimae*. Although *Vigna angularis* reference genome is available and useful for genetic analysis by genotyping-by-sequencing/RADseq in closely related species, it is not enough for isolation of responsible genes. To reveal sequence variation in *Vigna nakashimae* “Ukushima” and “G418”, the whole genome sequencing was performed using Illumina HiSeq X Ten system (411,174,986 and 478,116,282 read). NGS data was deposited in the DNA Data Bank of Japan (DDBJ) under accession number DRA009307.

**Table:** Specifications table

Subject area	Biology
Specific subject area	Agricultural and Biological Sciences, Genomics
Type of data	Genome sequence data
How data was acquired	Whole genomes of *Vigna nakashimae* ‘Ukushima’ and ‘G418’ were sequenced with ILLUMINA HiSeq X Ten sequencer.
Data format	Raw sequencing reads (fastq)
Parameters for data collection	Two salt tolerant strains of *Vigna nakashimae* ‘Ukushima’ and ‘G418’ were used for this study. They have been maintained in gene bank in NARO (https://www.gene.affrc.go.jp/databases_en.php). Genomic DNA for the sequencing was prepared from new leaves of one individual, respectively. Both accessions were purified using single seed descent method. Ukushima was self-pollinated more than 5 times since collection, and G418 was 2 times since collection in 2012.
Description of data collection	Sequencing libraries were prepared with 1 μg DNA input, using the TruSeq DNA PCR-Free Library Preparation Kit (Illumina). The library pools were quantified by qPCR, loaded on the HiSeq X patterned flow cells and clustered on an Illumina cBot following manufacturer's protocol. Flow cells were sequenced on the Illumina HiSeq X with 2 × 150bp reads. Demultiplexing of sequencing data was performed with bcl2fastq2.
Data source location	Tsukuba, Japan
Data accessibility	The sequence data have been deposited in the DNA Data Bank of Japan (DDBJ) Sequence Read Archive, under submission ID DRA009307 http://trace.ddbj.nig.ac.jp/DRASearch/submission?acc=DRA009307 (BioSample accessions: SAMD00193934 and SAMD00193935)

**Table:** 

**Value of the Data**
• Genomic data of high salt tolerance strains of *Vigna nakashimae* can be useful for detecting quatitative trait loci (QTL) or isolating genes.
• The data will give insight into salt tolerance in *Vigna nakashima*e.
• The data will help to understand the differentiation between cultivated Adzuki bean (*Vigna angularis*) and closely wild relatives, *Vigna nakashimae*.

## 1 Data description

To identify promising gene(s) source to improve salt tolerance of azuki bean, we have screened 69 azuki bean landraces together with cross compatible wild relatives consist of 150 accessions in 8 species [1,2]. We performed the primary screening in soil culture and the secondary screening in hydroponic culture. In this screening, we could not find good source of salt tolerance from azuki bean landraces. On the other hand, ‘Ukushima’ of *V. nakashimae* was identified as one of the most promising source of salt tolerance. In addition, we obtained more salt-tolerant stain ‘G418’ of *V. nakashimae* from the Goto Islands, Nagasaki, Japan [3,4].

We present whole genome sequence data of two salt tolerance *V. nakashimae* strains. We sequenced paired-end libraries using Illumina HiSeq X and generated approximately 411 and 478 M reads. The average percentage of Q30 bases (bases with a quality score of 30) and mean quality score were 89.56% and 90.49% for all reads, respectively. This data will be useful to build reference genome for genetic mapping using NGS (2nd generation sequencing) by genotype-by-sequence/RADseq instead of Adzuki bean (*Vigna angularis*) reference genome [5]. Additionally, this data will also be applicable for the diversity analysis of *Vigna* species using Adzuki genome in public [5].

## 2 Experimental design, materials and methods

### 2.1 Plant materials

In 2013, *Vigna nakashimae* ‘Ukushima’ (JP107879) and ‘G418’ (JP247291) plants were cultivated in a greenhouse at the National Agriculture and Food Research Organization in Tsukuba, Ibaraki, Japan. ‘Ukushima’ was identified in Yoshida et al. (2015).

### 2.2 DNA extraction and quality control

Genomic DNA was extracted from the newest leaf of *Vigna nakashimae* ‘Ukushima’ and ‘G418’ using CTAB method [6]. The quality of extracted DNA was determined by Nanodrop spectrophotometer (ThermoFisher Scientific, USA). In addition, the concentration of the DNA was determined using Qubit2.0 fluorometer (ThermoFisher Scientific, USA).

### 2.3 Library preparation and sequencing

A total of 411,174,986 and 478,116,282 paired reads of a 350-bp insert-size library by TruSeq DNA PCR Free kit (Illumina, San Diego, CA) were generated from Illumina HiSeq X from *Vigna nakashimae* ‘Ukushima’ and ‘G418’. The sequence data was deposited in the Sequence Read Archive (SRA) (biosample accession number: SAMD00193934 and SAMD00193935) under the bioproject accession number DRA009307.

## References

[1] Tomooka N., Isemura T., Naito K., Kaga A., Vaughan D., Singh M., Bisht I.S., Dutta M. (2014). *Vigna* species. Broadening the Genetic Base of Grain Legumes.

[2] Yoshida Y., Marubodee R., Ogiso-Tanaka E., Iseki K., Isemura T., Takahashi Y., Muto C., Naito K., Kaga A., Okuno K., Ehara H., Tomooka N. (2016). Salt tolerance in wild relatives of adzuki bean, *Vigna angularis* (Willd.) Ohwi et Ohashi. Genet. Resour. Crop Evol..

[3] Tomooka N., Fukui K., Chankaew S., Iizumi T., Hirashima S. (2013). Collection of wild Leguminous Crop relatives on Goto islands, Nagasaki, Japan.

[4] Ogiso-Tanaka E., Chankaew S., Marubodee R., Sakai H., Naito K., Baba A., Iseki K., Takahashi Y., Tomooka N. (2015). Identification of salt tolerant accessions in *Vigna nakashimae* and their genetic diversity analysis using RADseq. Breed Res..

[5] Sakai H., Naito K., Ogiso-Tanaka E., Takahashi Y., Iseki K., Muto C., Satou K., Teruya K., Shiroma A., Shimoji M., Hirano T., Itoh T., Tomooka N. (2015). The power of single molecule real-time sequencing technology in the de novo assembly of a eukaryotic genome. Sci. Rep..

[6] Chankaew S., Isemura T., Naito K., Ogiso-Tanaka E., Somta P., Kaga A., Tomooka N., Vaughan D.A., Srinives P. (2014). QTL mapping for salt tolerance and domestication-related traits in *Vigna marina* subsp. oblonga, a halophytic species. Theor. Appl. Genet..

